# Nuclear pore and nucleocytoplasmic transport impairment in oxidative stress-induced neurodegeneration: relevance to molecular mechanisms in Pathogenesis of Parkinson’s and other related neurodegenerative diseases

**DOI:** 10.1186/s13024-024-00774-0

**Published:** 2024-11-23

**Authors:** Zainab Riaz, Gabriel S. Richardson, Huajun Jin, Gary Zenitsky, Vellareddy Anantharam, Arthi Kanthasamy, Anumantha G. Kanthasamy

**Affiliations:** Isakson Center for Neurological Disease Research, Department of Physiology and Pharmacology, University of Georgia, Athens, GA USA

**Keywords:** Parkinson’s disease, Nuclear pore complex, Nucleocytoplasmic transport, Oxidative stress, Alpha-synuclein, Neurodegeneration

## Abstract

Nuclear pore complexes (NPCs) are embedded in the nuclear envelope and facilitate the exchange of macromolecules between the nucleus and cytoplasm in eukaryotic cells. The dysfunction of the NPC and nuclear transport plays a significant role in aging and the pathogenesis of various neurodegenerative diseases. Common features among these neurodegenerative diseases, including Parkinson’s disease (PD), encompass mitochondrial dysfunction, oxidative stress and the accumulation of insoluble protein aggregates in specific brain regions. The susceptibility of dopaminergic neurons to mitochondrial stress underscores the pivotal role of mitochondria in PD progression. Disruptions in mitochondrial-nuclear communication are exacerbated by aging and α-synuclein-induced oxidative stress in PD. The precise mechanisms underlying mitochondrial impairment-induced neurodegeneration in PD are still unclear. Evidence suggests that perturbations in dopaminergic neuronal nuclei are linked to PD-related neurodegeneration. These perturbations involve structural damage to the nuclear envelope and mislocalization of pivotal transcription factors, potentially driven by oxidative stress or α-synuclein pathology. The presence of protein aggregates, pathogenic mutations, and ongoing oxidative stress can exacerbate the dysfunction of NPCs, yet this mechanism remains understudied in the context of oxidative stress-induced PD. This review summarizes the link between mitochondrial dysfunction and dopaminergic neurodegeneration and outlines the current evidence for nuclear envelope and nuclear transport abnormalities in PD, particularly in oxidative stress. We highlight the potential role of nuclear pore and nucleocytoplasmic transport dysfunction in PD and stress the importance of systematically investigating NPC components in PD.

## Introduction

A vital characteristic of eukaryotic cells is compartmentalization of genomic DNA and specific cellular functions within the nucleus, which is enclosed by two concentric lipid bilayer membranes known as the nuclear envelope (NE). Regulation of gene expression and the cell cycle in eukaryotic cells relies on the continuous exchange of macromolecules between the nucleus and cytoplasm. This transport is facilitated by the nuclear pore complexes (NPCs) that are embedded throughout the NE [1]. NPCs are large macromolecular assemblies, of about 120 MDa in humans, that are embedded in the NE at sites where outer and inner nuclear membranes fuse to form channels [2]. These complexes are multifunctional; they serve as gatekeepers of the nucleus by forming a tightly regulated permeability barrier and are also key regulators of several crucial nuclear processes [3, 4].

The turnover of specific nucleoporins (Nups), the NPC building blocks, is very limited in quiescent cells, and certain components of the NPC, and its overall structure, can last throughout the lifespan of a cell [5]. This suggests that NPCs may accumulate damage and likely degrade over the course of normal and pathological aging [6, 7]. The loss of cellular compartmentalization is particularly detrimental to neurons, which are post-mitotic and highly polarized, and increasing evidence points to defective NPCs and nucleocytoplasmic transport (NCT) machinery in the pathophysiology of neurodegenerative diseases. More precisely, NE abnormalities, loss of specific Nups from the NPC, and cytoplasmic aggregates of Nups along with nuclear transport receptors have been observed in neurodegeneration [8, 9]. Cellular oxidative stress associated with aging and neuronal degeneration has also been implicated in nuclear pore deterioration [10]. Tissues that have long lifespans and are highly susceptible to oxidative stress such as dopaminergic (DAergic) neurons, which are pivotal to Parkinson’s disease (PD) pathology, may be more prone to NPC damage. In this review, we explore in detail the current literature that provides evidence for NE abnormalities and mislocalization of proteins in PD pathogenesis. Studies on NPC dysfunction in both oxidative stress and other neurodegenerative diseases have already been extensively reviewed, so we only briefly summarize them here in relation to the pathogenic mechanisms involved in PD. Importantly, we highlight the open questions regarding the potential role of NPC and NCT dysfunction in PD and emphasize the need to systematically investigate NPC components in PD.

## Nuclear pore complex structure and function

**Fig. 1:**
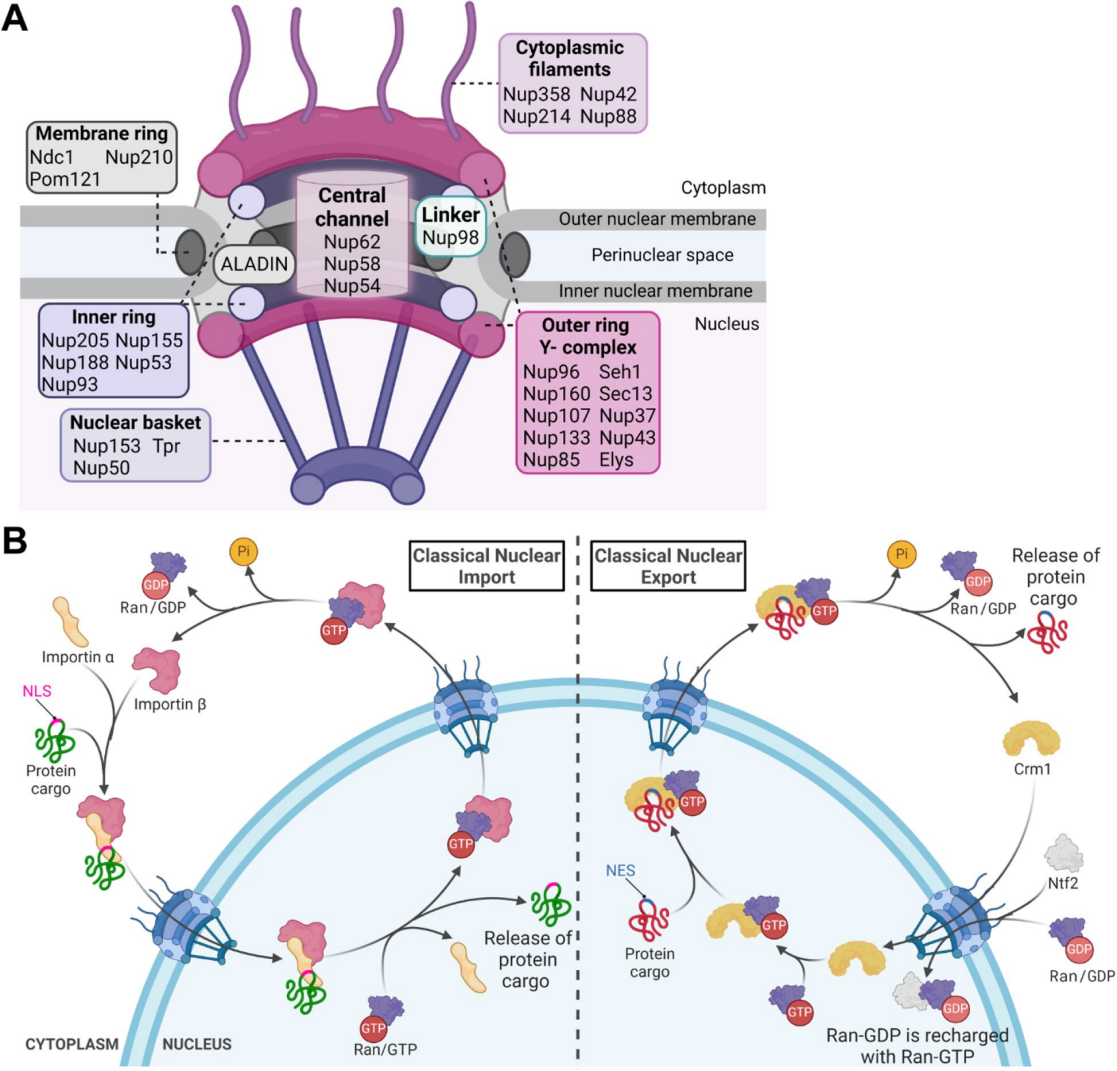
Illustrations of the nuclear pore complex and classical nucleocytoplasmic transport. **A** Structure of the nuclear pore complex showing the main subcomplexes and their component nucleoporins (Nups). *Created with BioRender.com*. **B** In classical nuclear import, protein cargo containing the nuclear localization signal (NLS) is transported into the nucleus by importins. In classical nuclear export, protein cargo containing the nuclear export signal (NES) is exported from the nucleus by the exportin Crm1. The directionality of nucleocytoplasmic transport is mediated by Ran GTPase. After each nuclear export cycle, Ran-GDP is shuttled back to the nucleus by nuclear transport factor 2 (Ntf2). Adapted from “TRPS1 Transport into the Nucleus” , created by “Joshua Patrick” using BioRender.com (2024). Retrieved from https://app.biorender.com/biorender-templates.

The NPC is made up of multiple copies of about 30 different Nups that assemble into stable subcomplexes [11]. These subcomplexes are comprehensively organized into a large protein complex characterized by eight spokes and eight-fold rotational symmetry along the axis perpendicular to the NE. The innermost components of the NPC form a core scaffold structure that is embedded in the NE and surrounds the central channel. This central channel connects the nucleoplasm to the cytoplasm and allows nucleocytoplasmic exchange of macromolecules [12]. The scaffold consists of three concentric rings: a membrane ring surrounded by two inner rings, and two outer rings that surround the inner rings and stabilize the curve in the NE. The two outer rings facing the nucleus and cytoplasm are called the nuclear and cytoplasmic rings, respectively [13]. The Nups within the core scaffold make up approximately half of the total NPC mass and are the most stably incorporated components of the NPC [14]. Asymmetric peripheral elements are attached to the core scaffold, and they line the central channel and protrude into the cytoplasm and nucleus. The Nups that fill the central channel contain multiple phenylalanine-glycine (FG) repeats and are referred to as FG-Nups, and they act as a selective barrier for nucleocytoplasmic transport (NCT) across the pore. Peripheral Nups attached to the cytoplasmic side of the core form cytoplasmic filaments, while those on the nuclear side create a nuclear basket structure [12] (Fig. 1A). Overall, FG-Nups represent one-third of the NPC components, and the unfolded structure of their FG domains interacts with nuclear transport factors [15, 16]. These Nups can undergo phase separation to form liquid condensates due to the dynamic hydrophobic crosslinking between FG motifs [17, 18].

### Scaffold Nups

The inventory of scaffolding domains adopted by these Nups is relatively limited and consists of α-solenoid and β-propeller folds, and an ancestral coatomer elements (ACE) domain. These protein folds are used in a modular arrangement to form subcomplexes in the NPC scaffold rings [19]. A key component of the outer rings is the Y-complex, also known as the Nup107-Nup160 complex. In humans, 16 copies each of the Y-complex, in the form of rotationally staggered self-dimers, make the nuclear and cytoplasmic outer rings. The structure of the Y-complex has been well characterized over the years and its name derives from its characteristic ‘Y’ shape [12, 20]. This subcomplex is evolutionarily conserved, but its Nup composition can vary between species. Seven conserved members of the Y-complex are recognized: Nup96, Nup160, Nup107, Nup133, Nup85, Sect. 13 homolog 1 (Seh1) and Sect. 13. In vertebrates, Nup37, Nup43 and Elys also bind to the Y-complex, with Elys binding only to the nuclear face of the NPC [20]. Three-dimensional reconstruction of the Y-complex using high-resolution crystal structures of individual components uncovered the modularity and relative positions of individual Nups in this subcomplex [21]. The Y-complex has been shown to be crucial for post-mitotic assembly of the NPC [22, 23]. Depletion of this subcomplex in vitro leads to the formation of nuclei with a continuous NE that lacks NPCs. NPCs were assembled in these cells if the Y-complex was added back before NE formation [23]. The inner ring of the scaffold is the most structurally conserved component of the NPC. Inner ring Nups anchor the NPC scaffold to the NE membrane and serve as attachment points for the central channel FG-Nups [24]. Inner ring Nups comprise Nup205, Nup188, Nup93, Nup155, and Nup53 in vertebrates [13]. Nup53 and its interaction with Nup155 is essential for NE formation and NPC assembly [25]. Recent modeling of individual Nups and NPC subcomplexes has suggested that two copies of Nup93 form a bridge between the Y-complexes on nuclear and cytoplasmic rings [26]. To identify the most critical Nups for NPC assembly, Sakuma et al. [27] used a small interfering RNA (siRNA) screen against 28 Nups to show that depletion of components from the Y-complex and the inner ring Nup93-205 complex most strongly impairs NPC formation. The inner ring is attached to the pore membrane by interaction with the Nups in the membrane ring. Transmembrane Nups include Ndc1 (Nuclear-Division-Cycle 1), Pom121 (Pore membrane protein of 121 kDa), and Nup210. Ndc1 is highly conserved across species, while Pom121 is the least conserved transmembrane Nup and is unique to vertebrates [28]. These Nups have transmembrane helical domains and they tether and secure the NPC to the NE [29]. Ndc1 is involved in localization of the inner ring Nup53 to the NE and is therefore required for NPC assembly [30]. Ndc1 also interacts with and anchors another nuclear pore component, ALADIN, which is involved in nuclear import to the NE [31]. Pom121 interacts with the inner nuclear membrane proteins Lamin B receptor and Sun1 (of the linker of nucleoskeleton and cytoskeleton or LINC complex) and plays a crucial role in NPC assembly [32, 33]. During interphase NPC assembly, Pom121 also associates with the inner ring Nup155 and outer ring Nup160, which may facilitate incorporation of the Y-complex as an early event in NPC assembly. While the N-terminal segment of Pom121 is predicted to interact with Nups155 and 160, the C-terminal region of this protein contains several FG repeats and it may protrude beyond the scaffold coat and into the NPC central channel [34]. Pom121 depletion severely reduced NPC numbers, which is shown by loss of the fluorescence signal from the NPC marker antibody MAb414, which binds to several FG-Nups [28].

### Central channel Nups

Immunoelectron microscopy has placed three mammalian Nups at the NPC central channel: Nup62, Nup58, and Nup54 [35]. These Nups form the trimeric Nup62 subcomplex, which is connected to the NPC scaffold by direct coiled-coil interaction with the inner ring Nup93 [36]. One copy of the Nup62 complex is attached to each inner ring complex, thereby concentrating the corresponding FG domains at the equatorial plane of the NPC [37]. The Nup62-Nup58-Nup54 trimeric complex is essential for nuclear transport [38], and Nup62 is shown to directly interact with the nuclear transport receptor, importin β [39].

Overall, FG-Nups represent one-third of the NPC components, and the unfolded structure of the FG domain interacts with nuclear transport factors [15, 40]. The FG repeat clusters are predominantly present as Phe-Gly (FG), Gly-Leu-Phe-Gly (GLFG), or Phe-any-Phe-Gly (FxFG) [41]. FG-Nups in the central channel are anchored to the NPC via an alpha-helical structured domain, and the unstructured FG domain extends into the channel [42]. How the central channel FG-Nups form a selectively permeable barrier for NCT has been a subject of great interest in NPC research, and while several models have been proposed for it, the precise mechanism remains unclear. The most plausible and well-supported model is the selective phase model, which suggests that the FG repeats extend into the central channel and interact to form a mesh that facilitates the selective transport of cargo attached to the nuclear transport receptor [43]. Indeed, Frey et al. [44] have shown that yeast FG-Nups form inter-repeat interactions that cross-link the FG domains into elastic and reversible hydrogels. In another study, NPCs reconstituted from *Xenopus laevis* egg extracts were used to show that cohesion between FG repeats forms a sieve-like hydrogel, which is required to maintain the NPC permeability barrier. Specifically, this study highlighted the role of the highly cohesive GLFG repeat containing Nup98 in maintaining the barrier [45]. Nup98 is highly mobile, and it serves as a flexible linker of outer and inner ring structures in the NPC scaffold, while its hydrophobic FG motifs contribute to the permeability barrier [46, 47]. Nup98 also directly interacts with the exportin Crm1 (Chromosomal Maintenance 1) in a Ran GTPase-dependent manner and serves as a shuttling cofactor in nuclear export [48]. It has recently been shown that more than half of the FG-Nups can undergo phase separation to form liquid condensates due to the dynamic hydrophobic crosslinking between FG motifs. Importantly, central channel FG-Nups primarily contain GLFG repeats, which typically form a remarkably dynamic network through numerous short-lived FG-FG interactions. Peripheral FG-Nups are mainly of the FxFG type and are predicted to form an ectopic barrier at the entry and exit of the nuclear pore [17].

### Nuclear basket Nups

The peripheral structure of the NPC in the nuclear compartment is termed the nuclear basket. This structure consists of eight filaments that are attached to the nuclear outer ring and form a distal ring in the nucleoplasm [37]. The nuclear basket comprises Tpr [49], Nup50 [50], and Nup153 [51]. Nuclear basket Nups contain FG-repeats, α/β motifs, α-helical domains, and intrinsically disordered regions [52]. Tpr is the central architectural component that forms the core structure of the nuclear basket [53]. Tpr has an intrinsically disordered C terminal with the lowest number of FG repeats among FG-Nups, and N terminal coiled-coils [54]. It has been reported that Tpr interacts with Crm1, and that depletion of Tpr compromises nuclear export signal (NES)-mediated export of proteins [55, 56]. Tpr also serves as a regulator of mRNA export via the mRNA export factor Nxf1 and it also facilitates tRNA export [54]. Another key function of Tpr is negative regulation of the NPC assembly through recruitment of the Y-complex to the NE. NPC numbers and density in the NE are determined by protein levels and phosphorylation status of Tpr [57].

Nup50 directly facilitates nuclear import by functioning as a co-factor with the nuclear import proteins importin α and importin β [58]. Nup50 associates with the nuclear basket via interaction of its N terminal region with Nup153, both directly and through importin α. The Nup153-Nup50 interface provides a platform for efficient NCT [59]. Structurally, Nup153 contains three domains: a unique N terminal domain that is pore-targeting, a central zinc finger region and a C terminal domain with several FxFG repeats [60]. The N terminal domain of Nup153 interacts with Tpr as well as the Y-complex in the nuclear coaxial ring, which may help in anchoring Tpr, and hence the nuclear basket, to the NPC core [53]. The C terminal FG domain is highly flexible and interacts with nuclear transport receptors, indicating its role in NCT [54]. Nup153 also binds to the nuclear lamina and Sun1 protein [61] of the LINC complex. Depletion of either Nup153 or lamin leads to NPC clustering, suggesting that this interaction may regulate the distribution of NPCs across the NE [62, 63].

### Cytoplasmic filament Nups

Peripheral Nups on the cytoplasmic face of the NPC form elongated structures called cytoplasmic filaments. Cytoplasmic filament Nups contain FG repeats, and like the nuclear basket Nups, they contribute to NCT by providing a docking site for macromolecular cargo and nuclear transport factors. Additionally, these Nups play a role in export and remodeling of messenger ribonucleoprotein particles (mRNPs) in preparation for translation [64]. Cytoplasmic filaments comprise three FG-Nups: Nup358, Nup214, and Nup42 [54]. Nup358 is only present in vertebrate NPCs and is the largest known Nup. The Nup358 complex, also known as the Ran-binding protein 2 (RanBP2) complex, is a prominent component of cytoplasmic filaments [65]. Structurally, Nup358 contains several distinct domains including FG repeats, binding sites for the small GTPase Ran, an E3 ligase domain, an S-shaped α-helical solenoid followed by a coiled-coil oligomerization element at the N terminal, and a C-terminal prolyl-isomerase domain. Near-atomic composite maps of the cytoplasmic filaments from advanced structural characterization show that pentameric complexes of Nup358 are linked by the oligomerization element and affixed to the central stalk region of the NPC through their N-terminal domains. This arrangement allows flexible attachment of domains that project far into the cytoplasm [64]. Nup358 is shown to be dispensable for architectural integrity of the NPC and export of RNA, but it is needed for efficient translation [64, 66]. A non-FG Nup, Nup88, also localizes to the cytoplasmic face of the NPC and interacts with Nup214. The coiled-coil regions of Nup214 and Nup88 also interact with the central channel FG-Nup Nup62 to form a heterotrimeric hub [12, 26]. The Nup214-Nup88 subcomplex facilitates nuclear export by directly binding to the nuclear export receptor Crm1 [67]. The FG domain in the extreme C terminal of Nup214 also interacts with the mRNA export protein Nxf1 [68]. Nup42 interacts with the shuttling mRNA export mediator Gle1 via a non-FG region [69]. The DEAD-box ATPase (Dbp5/DDX19) interacts with Nup214 [70] and also forms a complex together with Nup42 and Gle1 [71] to orchestrate mRNP remodeling in the final stages of mRNA export on the cytoplasmic side of the NPC. Gle1 also interacts with the C terminal of the inner ring Nup155 via its N terminal, and this association may help position Gle1 along the NPC in the mRNA export pathway [26]. Another shuttling mRNA factor, Rae1, contributes to mRNA export by forming an mRNP-binding complex with the mobile linker Nup98 at the cytoplasmic face of NPC [72].

### Conservation and dynamics of the NPC architecture

Being crucial gatekeepers of nuclei, NPCs are present in all eukaryotic cells and the overall eight-fold symmetry of these complexes is largely conserved across all species. While the NPC is functionally preserved across species, from yeast to humans, variations in the structural assembly of NPCs can be observed. Firstly, the mass of yeast and human NPCs substantially differ and the stoichiometry of certain Nups in yeast is lower when compared to the human pore complex. Integrative structural modeling of the NPC has revealed that the inner ring is smaller in *S. cerevisiae* compared to humans but the overall arrangement of Nups is similar in both. Secondly, the connection between inner and outer rings, which is formed by Nup155 in vertebrates, is missing in the yeast NPC [73, 74]. A remarkable difference emerged in the nuclear and cytoplasmic outer rings. Although the Y-complex is conserved, eight copies of this complex make up each ring in yeast, with a total of 16 copies of the Y-complex in each NPC. In humans, however, both nuclear and cytoplasmic rings are made of two concentrically arranged eight-membered rings of the Y-complex, with a total of 32 copies of the Y-complex in each NPC [75]. Interestingly, the algal NPC lacks two-fold symmetry along the NE axis because it has a single Y-complex ring on the cytoplasmic face but two concentrically arranged Y-complex rings on the nuclear side, with 24 Y-complex copies in total. Cryo-electron tomography reconstruction of the human NPC, derived from HeLa cells depleted of Nup358, revealed human NPCs with a singular outer cytoplasmic ring. Therefore, the differences in the stoichiometry of the cytoplasmic outer rings between these species may be explained by the absence of direct homologs of Nup358 in both *S. cerevisiae* and the unicellular algal species *C. reinhardtii*. Another difference in the algal NPC is the dilated inner ring, yielding a wider central channel [12].

NPCs have been considered as relatively static structures, unlike the dynamic shuttling of macromolecular cargo and transport receptors across the pores during NCT. However, studies on NPC dynamics show that, despite the stable embedding of NPCs in the NE, their subcomplexes and individual components may undergo dynamic modifications. These include conformational adjustments of the NPC during NCT and broader structural transitions throughout the cell cycle [76]. A cryo-electron tomography study [77] of NPC rearrangement during NCT revealed that cytoplasmic filaments curve toward the central channel, suggesting a possible direct interaction with cargo during the transport process. In another study [78] on spatiotemporal dynamics of the NPC, the highly flexible FG-Nups that surround the cytoplasmic opening of the NPC underwent rapid elongation and retraction. The study further revealed that the central channel FG-Nups display interweaving transient entanglements but do not form a rigid, tightly crosslinked meshwork. Consequently, the functional structure of the NPC barrier consists of highly dynamic FG-Nups, which appear as a central plug when averaged over space and time.

Individual Nups may be very dynamic and exhibit transient associations with the NPC. Hakhverdyan et al. [14] comprehensively investigated the dynamics of individual NPC components and found that turnover of Nups is uniformly slow but they are exchanged in the NPC at significantly different rates. The variation in Nup exchange correlated with their structural role rather than peripheral position in the NPC. The outer, inner, and membrane rings of the NPC scaffold are the most stably incorporated subcomplexes, apart from membrane ring component Ndc1. Similarly, core components of cytoplasmic filaments are also stable and have low exchange rates. The most dynamic NPC components are the flexible linker Nups and Nups involved in RNA export and RNA remodeling factors such as the nuclear basket and Gle1. This is in agreement with previous studies that have shown that scaffold Nups such as the Y-complex show stable association with the NPC while nuclear basket proteins like Nup153 and Nup50 exhibit highly dynamic behavior [79]. It has been shown that Nup107 and Nup93 are not exchanged once incorporated into the NPC [80]. Among dynamic Nups, Nup50 exhibits the shortest association time with the NPC [81]. It has been suggested that modular construction of the NPC and exchange of Nups ensures the maintenance of NPCs and imparts resilience to damage [14].

### Nucleocytoplasmic transport

The NPC forms a soft barrier to passive diffusion. While small molecules can pass freely through the pore, the FG domains near the cytoplasmic side of the central channel, characterized by high net charge and low hydrophobicity, hinder the diffusion of larger macromolecules [82]. Therefore, larger macromolecular cargoes must rely on soluble nuclear transport receptors for transport through the central channel of the NPC [15, 83]. These nuclear transport factors specifically identify and bind to cargo destined for translocation, efficiently shuttling them back and forth through the nuclear pores. The transport signals required for import into and export from the nucleus are respectively termed the nuclear localization signal (NLS) and nuclear export signal (NES) [40]. Nuclear transport receptors are divided into importins for nuclear entry and exportins for exit from the nucleus [84]. The directionality of NCT is determined by a concentration gradient of Ran GTPase across the nucleus. Nuclear Ran primarily exists in a GTP-bound state, while cytoplasmic Ran is GDP-bound [85]. This steep Ran GTPase gradient is maintained by the guanine nucleotide exchange factor (RCC1) in the nucleus and a GTPase-activating protein (RanGAP) in the cytoplasm. RCC1 is bound to chromatin within the nucleus, and it promotes the release of GDP from Ran by stabilizing nucleotide-free Ran in the nucleoplasm. In the cytoplasm, RanGAP and Ran-binding protein RanBP1 stimulate the hydrolysis of GTP to maintain cytosolic Ran in the GDP-bound state [86]. In classical nuclear import, NLS-containing protein cargo is recognized by importins, importin α and importin β, which guide the cargo to the NPC and physically associate with FG-Nups to translocate the import complex through the NPC central channel. In the nucleus, Ran-GTP interacts with the import complex and reduces the interaction between importins and cargo, causing complex disassembly and cargo release into the nucleus. Subsequently, Ran-GTP forms a complex with importin β, which is shuttled back to the cytoplasm for recycling [87]. In nuclear export, Ran-GTP cooperatively associates with the exportin Crm1 in the nucleus, which initiates the binding of NES-containing cargo with Crm1. The cargo-Crm1-Ran-GTP trimeric complex interacts with Nups and translocates through the NPC to the cytoplasm. Once on the cytoplasmic side, RanGAP induces the hydrolysis of GTP to GDP in Ran, leading to the simultaneous dissociation of Ran from Crm1 and the release of cargo into the cytoplasm. The cytoplasmic Ran-GDP associates with nuclear transport factor 2 for transport back into the nucleus through the NPC. Upon reaching the nucleoplasm, Ran-GDP is recharged with GTP, thereby replenishing nuclear Ran-GTP for another cycle of nucleocytoplasmic trafficking [88] (Fig. 1B). The export of bulk mRNA, however, is atypical and follows a distinct mechanism that requires a different set of export receptors and does not rely on the Ran-GTPase gradient [89]. The transcription-export (TREX) complex is assembled on completely processed mRNA, and comprises the multiprotein THO complex and several mRNA export factors including Nxf1-Nxt1 [90].

### Transport-independent role of Nups

In addition to their primary role as facilitators of the nucleocytoplasmic exchange of molecules, NPCs play a key role in regulating various cellular processes independent of transport. Increasing evidence shows the involvement of NPCs in genome organization and the regulation of gene expression through functional connectivity. Chromatin has been found to bind both stable and dynamic Nups, and Nup target genes are observed both at the nuclear periphery in proximity to NPCs and within the nucleoplasm where they are bound by soluble Nups [91, 92]. The initial evidence of NPCs playing a role in the activation of gene expression came from a study [93] in *S. cerevisiae* that showed involvement of the Nup84 (mammalian Nup107) subcomplex in transcriptional stimulation. In yeast, the movement of several genes from the nuclear interior to NPCs has been demonstrated upon activation, and it is evident that NPC-tethering plays a crucial role in maintaining proper gene expression and transcriptional memory [94]. Studies [91, 92] in *Drosophila* have interestingly shown that Nup50, Nup62, Nup88, Sect. 13, and Nup98 regulate the expression of developmental and cell cycle genes by directly interacting with gene loci in the nucleoplasm away from the NPC. They further noted that Nup88 interacts with silent loci but nucleoplasmic Sect. 13, Nup50, Nup62, and Nup98 primarily localized to transcriptionally active genes. NPC-tethered Nup98, however, was shown to induce gene silencing. In different human cell cultures, Nup153 and Nup93 bind to super-enhancers of a subset of cell-type-specific genes at the nuclear periphery [95]. Like Nup98, Nup153 also interacts with the genome away from the NPC in both *Drosophila* and mouse cells [96, 97]. Being FG-Nups, these Nups may undergo phase separation in the nucleoplasm to form foci. Such intranuclear Nup98 foci have been observed in some cell types and are shown to recruit additional Nups, such as components of the Y-complex [98].

Nups function as epigenetic regulators of gene expression in a cell type- and context-dependent manner by interacting with different protein partners such as transcription factors and chromatin-modifying proteins. Notably, several Nups are involved in regulating the expression of differentiation genes and are crucial in establishing the balance between cell stemness and differentiation [99].Moreover, a growing body of evidence suggests that Nups are involved in various functions within the central nervous system and significantly influence neurogenesis and neurophysiology by serving as key mediators of neuroepigenetics [100]. The NPC also confers stability to the genome by serving important functions in the DNA damage response pathways [101]. Specifically, Nup153 and Nup50 act in concert to import the key double-strand break repair factor 53BP1 into the nucleus and target it to damaged foci [102, 103].

## Parkinson’s disease

**Fig. 2:**
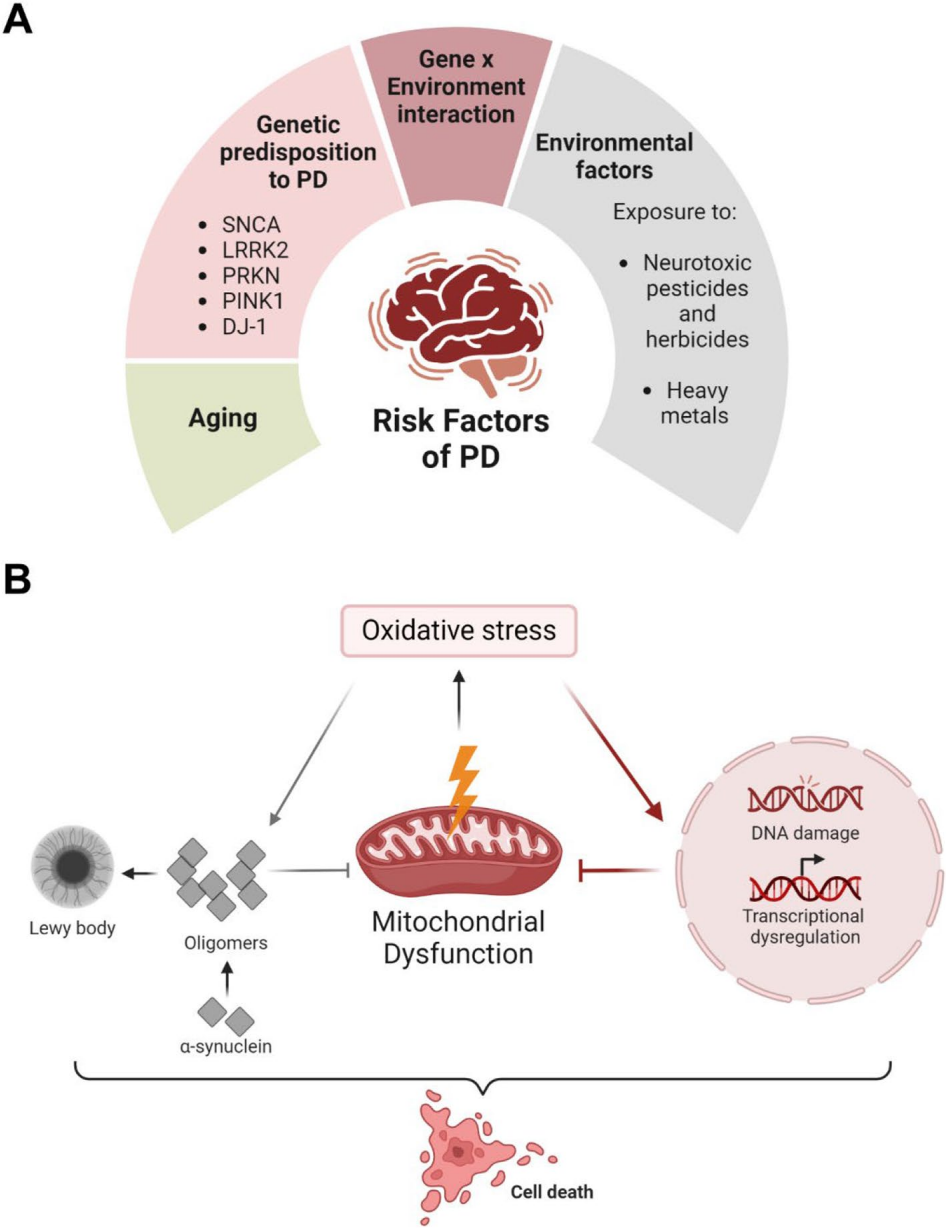
The risk factors and molecular pathways associated with PD. **A** A schematic overview of the etiology of PD. **B** The interplay between mitochondrial dysfunction, oxidative stress, α-synuclein toxicity, and aberrant mitochondria-nuclear signaling contributes to DAergic neuron loss in PD. *Created with BioRender.com*

The neuropathology of PD is characterized by a progressive loss of DAergic neurons within the substantia nigra (SN), leading to a deficit of striatal DA levels [104]. Another pathological hallmark of PD is the deposition of abnormal aggregates of α-synuclein protein within the cell bodies of neurons [105]. These intracytoplasmic aggregates are called Lewy bodies, and in addition to α-synuclein, they contain several other proteins like ubiquitin, heat shock proteins, tau, and proteasomal and lysosomal elements [106]. The cardinal motor symptoms associated with PD are tremors, rigidity, slowness of movement, and postural instability [105]. Several non-motor symptoms such as olfactory deficits, rapid eye movement sleep behavior disorder, autonomic changes and depression are also associated with PD, and these symptoms usually appear in the prodromal stage of the disease [107]. The etiology of PD is complex and multifactorial with gene-environment interactions playing key roles in disease development. Genetic mutations underlie a minority of PD cases. Approximately 20 different genes have been identified in familial PD studies, and genome-wide association studies have identified over 200 genes as potential risk factors in sporadic PD [108]. Environmental factors are believed to play an important role in the causation of most sporadic PD cases where consequential mutations in PD-linked genes are absent. Data from epidemiological studies regarding environmentally linked PD has been inconsistent, making causal inference difficult [109]. Nonetheless, occupations with a higher PD risk have been investigated, and these include working with pesticides [110, 111] and heavy metals [112]. A primary risk factor for idiopathic PD is aging, with the incidence of PD increasing in individuals over 65 years of age [113, 114] (Fig. 2A).

### Mitochondrial dysfunction and the selective vulnerability of DAergic neurons

Several lines of evidence have implicated mitochondrial dysfunction in DAergic neurodegeneration in PD. Mitochondrial impairment was directly linked to PD by the observation that complex I subunits and activity are significantly reduced in the SN and striata of PD patient brains compared to healthy controls [115, 116]. Keeney et al. [117] reported increased oxidation and the misassembly of mitochondrial complex I subunits as well as reduced rates of electron transfer through complex I in PD brains. A recent proteomic analysis of the human PD brain by Toomey et al. [118] demonstrated that mitochondrial dysfunction is present across multiple brain regions associated with Braak staging. Mitochondrial impairment occurred prior to or simultaneously with α-synuclein aggregation, before neuronal loss emerged, and is thus implicated as an early driver of sporadic PD. The common pathways that were altered in the PD brains include oxidative phosphorylation and ATP synthesis, mitochondrial metabolism, and glutathione metabolism and redox regulation. Chen et al. [119] studied multiple protein targets in post-mortem brains of PD patients and control cases using imaging mass cytometry. Their analysis revealed a significant reduction in mitochondrial quality control machinery in the SN of PD brains, suggesting disrupted mitochondrial turnover and proteostasis. DAergic neurons have elevated energetic requirements because of their exceptionally long axonal arbor and autonomous pacemaking [120–122]. Moreover, DAergic neurons produce and are exposed to oxidative stress because of DA metabolism, particularly its degradation and oxidation, which generate reactive oxygen species (ROS) and reactive DA quinones, respectively [122, 123]. These data show that DAergic neurons are particularly susceptible to mitochondrial dysfunction; however, the precise mechanisms underlying this selective vulnerability are poorly understood.

Mitochondria are a common target of many genetic mutations and environmental toxicants associated with PD [124]. Mutations in several PD-linked genes, including *SNCA*, *LRRK2*, and *DJ1* have been shown to induce mitochondrial stress and increased ROS production [125–127]. LRRK2 is a multifunctional protein that affects mitochondrial function, and mutations in its gene are a prevalent cause of late-onset autosomal dominant PD [128]. Most common PD-associated mutations in *LRRK2* disrupt calcium homeostasis, impair mitophagy and mitochondrial fission and fusion, and lead to increased oxidative stress [129]. *PINK1* and *PRKN* are the most common autosomal recessive genes associated with early-onset PD. The products of these genes function in concert with the mitophagy pathway and their PD-associated mutations therefore impair mitochondrial quality control [130, 131]. The initial evidence that connected mitochondrial impairment to PD came from unintentional exposure of illicit drug users to the mitochondrial complex I inhibitor MPTP (1-methyl-4-phenyl-1,2,3,6-tetrahydropyridine). Exposed individuals developed irreversible and progressive Parkinsonism and DAergic neurodegeneration [132, 133]. Epidemiological studies have identified a positive association between PD risk and exposure to pesticides like rotenone or paraquat that are known for either inhibiting mitochondrial complex I or inducing oxidative stress [134–136]. Neurotoxins like MPTP, rotenone, 6-hydroxydopamine (6-OHDA), and other mitochondria-impairing toxins are shown to induce DAergic neuron loss in animal models, allowing them to exhibit the clinical features of PD. This approach is widely used to create experimental animal and cell models that mimic sporadic PD to investigate the underlying mechanisms [137, 138]. Heavy metals such as iron and manganese also contribute to PD progression by inducing free radical production, oxidative stress and mitochondrial dysfunction [139].

### Crosstalk between mitochondrial dysfunction, oxidative stress and α-synuclein toxicity

In aging individuals, as critical processes essential for DAergic neuron function begin to decay, the accumulation of ROS and oxidative stress from DA metabolism also accelerates. The combined effect of these age-related defects renders these neurons susceptible to additional stressors such as mitochondrial complex I impairment and toxic α-synuclein species [140]. Interestingly, evidence is emerging for considerable crosstalk between mitochondrial dysfunction, oxidative stress and α-synuclein toxicity pathways in PD. Both mutant and wild-type α-synuclein have been found to localize to mitochondrial compartments, where they interact with mitochondrial proteins like Parkin, PINK1 and ATP synthase and regulate mitochondrial dynamics and bioenergetics [141, 142]. While monomeric α-synuclein enhanced ATP production [141], α-synuclein oligomers and overexpression of both wild-type and mutant α-synuclein decreased complex I activity, increased ROS production and contributed to mitochondrial dysfunction [143–145]. In a recent study, Choi et al. [146] tracked the intracellular conformational states of A53T mutant α-synuclein and revealed that its seeding events predominantly take place on membrane surfaces, particularly on mitochondrial membranes, and the mitochondrial lipid cardiolipin triggers A53T α-synuclein oligomerization. Another study [147] has shown that pathological α-synuclein aggregates, but not overexpressed α-synuclein alone, exhibit transient and dynamic interactions with mitochondria, resulting in mitochondrial depolarization, reduced ATP production, and mitochondrial fragmentation and degradation. The aggregation of α-synuclein also correlates with diminished mitochondrial content in human DAergic neurons and the midbrain of mouse models. Similarly, mitochondrial dysfunction and oxidative stress can affect α-synuclein accumulation and pathology by oxidative modifications and misfolding of α-synuclein, making it partly resistant to proteasomal degradation [148, 149]. Therefore, an intimate connection exists between mitochondrial dysfunction and α-synuclein toxicity in the progression of DAergic neuronal loss. More importantly, the evidence for the vulnerability of DAergic neurons to mitochondrial toxins, mitochondrial alterations found in post-mortem PD tissue, and the association of mutations in mitochondrial proteins to PD risk shows a crucial pathogenic role for mitochondria in PD progression. Furthermore, evidence of oxidative DNA damage in PD brains was put forth by Dias et al. [150, 151]. Conversely, mitochondrial biogenesis is dependent on organized transcriptional activity of both mitochondrial and nuclear genomes [119]. Aberrant mitochondrial-nuclear communication and impaired nuclear transcriptional regulation of mitochondrial proteins also contribute to the decline in mitochondrial turnover that occurs in aged and PD neurons. This may worsen the effect of aging-related and α-synuclein-induced oxidative stress (Fig. 2B). We must enhance our understanding of the precise mechanisms by which mitochondrial impairment contributes to the genetic and sporadic manifestations of PD if effective mitochondria-based therapies are to be developed [121].

### Evidence of altered nucleocytoplasmic transport in PD

**Fig. 3:**
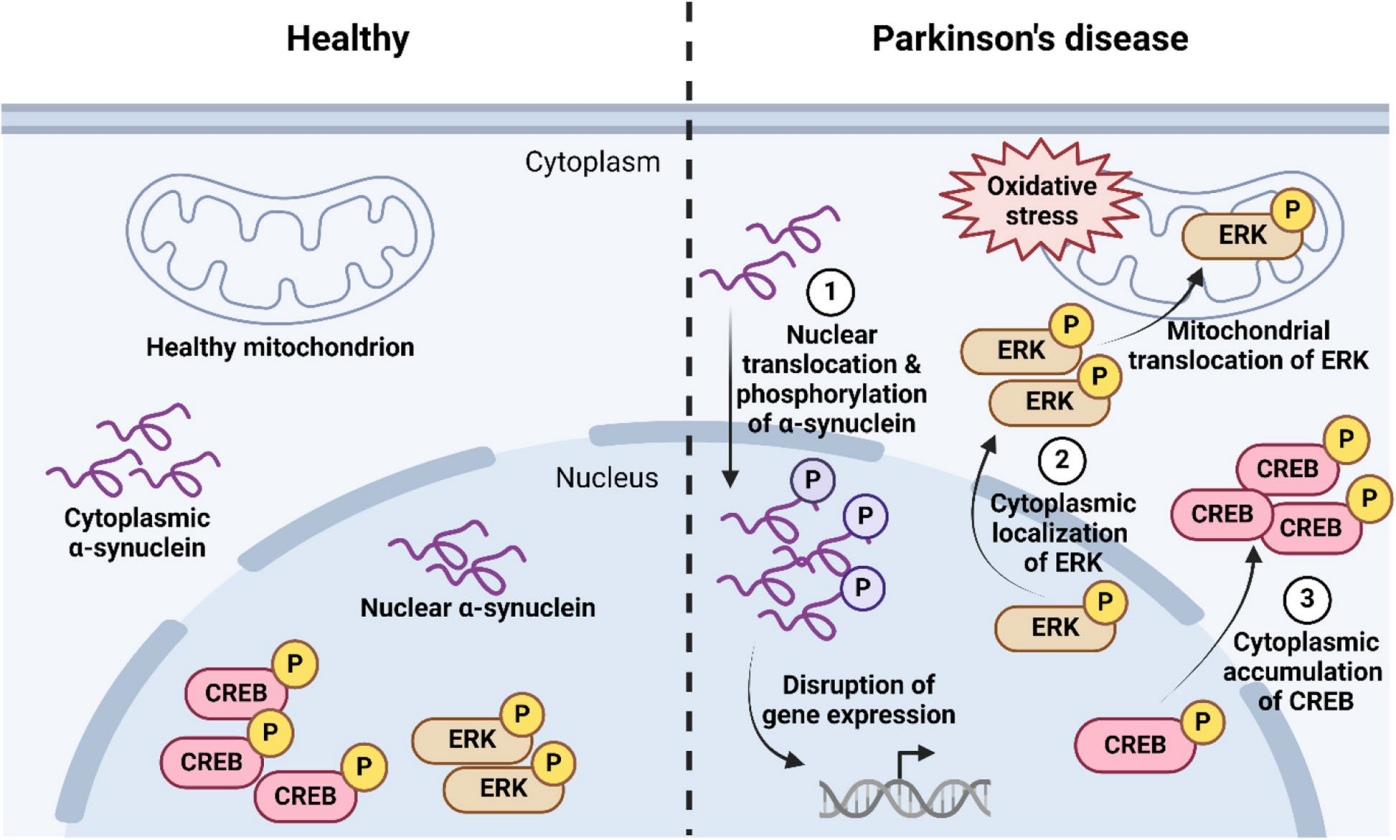
Summary of mislocalization of specific proteins in DAergic neurons in PD. (1) Oxidative stressors increase the translocation of α-synuclein to the nucleus where it can markedly alter gene expression. This could account for the accumulation of phosphorylated α-synuclein in the nuclei of patient brains. (2) Phosphorylated extracellular signal-regulated kinase (ERK) and (3) total and phosphorylated cyclic AMP response element-binding protein (CREB) accumulate in the cytoplasm of dopaminergic neurons exposed to 6-hydroxydopamine and in PD patient neurons. A fraction of the cytoplasmic phospho-ERK also localizes to the mitochondria. *Created with BioRender.com*

Multiple studies have shown the mislocalization of specific proteins such as transcription factors in PD, implicating NCT defects in disease pathogenesis. In primary DAergic neurons exposed to the oxidative neurotoxin 6-OHDA, total and phosphorylated cyclic AMP response element-binding protein (CREB) accumulate in the cytosol, accompanied by their decreased nuclear staining. Moreover, cytoplasmic phospho-CREB increases in degenerating nigral neurons of PD and Lewy body disease patients compared to healthy controls [152]. In neurons, CREB activity has been associated with diverse physiological processes such as cell proliferation, survival, differentiation, plasticity, and synaptic activity [153]. Therefore, mislocalization of CREB may lead to an altered transcriptional cascade, which is likely to have significant impacts on neuronal viability and function. Similarly, granulated immunoreactivity of phosphorylated extracellular signal-regulated kinase (ERK) has been observed in the cytoplasm of nigral neurons with Lewy body pathology in PD brain tissues [154, 155]. Phospho-ERK alteration appeared at an earlier disease stage. Exposure of an in vitro neural cell culture model to 6-OHDA also induced cytoplasmic localization of phospho-ERK, which was blocked by treatment with the antioxidant catalase, indicating the role of oxidative stress in phospho-ERK alteration. Aberrant cytoplasmic localization of catalytically active ERK alters the downstream targets, which may have important implications for disease pathogenesis [154]. A fraction of the cytoplasmic activated ERK also localizes to the mitochondria and induces mitophagy and autophagic stress during neurodegeneration [156] (Fig. 3). In a forward genetics screen to identify regulators of gene expression in presynapses, the THO nuclear export complex was found as a crucial regulator of synaptic mRNA export in DAergic neurons. THO complex mutations in *C. elegans* induce entrapment of synaptic transcripts in the nucleus, leading to decreased protein expression and impaired DAergic presynapses. Conditional deletion of THO complex subunit 5 in mouse DAergic neurons leads to severely compromised synapse maintenance, DAergic neurodegeneration, and locomotor defects [157]. These findings suggest that dysfunction of the THO complex and nuclear mRNA export has crucial implications for DAergic neuronal function.

Recent progress in understanding the underlying molecular mechanisms of neurodegenerative disorders has linked the mislocalization and abnormal accumulation of disease-causing proteins to impaired NCT [158]. Oxidative stressors increase the nuclear translocation of α-synuclein in DAergic neurons in vitro and in vivo [159, 160]. Moreover, while nuclear α-synuclein has been found in both control and dementia with Lewy body disease brain tissues, phosphorylated α-synuclein shows a 14-fold increase in the nuclei of patient brains [161]. Nuclear localized α-synuclein promotes neurotoxicity by altering transcription, histone modification and inducing DNA damage [159, 162]. Nuclear α-synuclein also exacerbates mitochondrial dysfunction by transcriptional modulation of the mitochondrial regulator, PGC1-α [163] (Fig. 3). Synucleinopathies have been associated with abnormalities in nuclear transport receptors [164]. In a recent study, mice were engineered to express endogenous α-synuclein fused with an NLS. These mice show age-dependent motor deficits, motor cortex atrophy, and dysregulation of proteins involved in dopaminergic signaling. Notably, these phenotypes appeared in the absence of α-synuclein phosphorylation or aggregation, indicating that the chronic buildup of nuclear α-synuclein can induce toxic phenotypes in mice [165]. Phosphorylation [166], cleavage [160], and interaction with other nuclear proteins [167, 168] are suspected in the nuclear mislocalization of α-synuclein, but the exact factors and cellular mechanisms that induce nuclear accumulation of α-synuclein in PD are unclear. A specific NLS is lacking in α-synuclein, so it is unclear whether its nuclear uptake involves active or passive mechanisms, or how α-synuclein is retained in the nucleus. It was suggested that nuclear import of α-synuclein may be mediated by importin α [169], but more recently Chen et al. [167] have demonstrated that the downregulation of Ran has no impact on the nuclear localization of α-synuclein. Thus, the nuclear import of α-synuclein may be passive. Further studies are needed to elucidate the mechanism of nuclear import and export of α-synuclein and its interaction with the nuclear pore components.

### PD-linked mutations disrupt the nuclear envelope architecture

Another potential pathological mechanism underpinning DAergic neurodegeneration in PD that is worthy of further investigation is the impairment of NPC components and the NCT apparatus. To date, only a handful of studies have investigated nuclear membrane alterations in PD, however, PD-linked mutations are known to disrupt the NE architecture. Studies on the mechanistic role of nuclear α-synuclein show that A53T and triplication mutations of the *SNCA* locus exacerbate nuclear aging and disrupt NE integrity in stem cell-derived DAergic neurons [167, 170]. Furthermore, α-synuclein is involved in NCT by interaction with Ran GTPase, and A53T mutant α-synuclein aberrantly sequesters Ran preventing it from orchestrating the transport of macromolecules across the nucleus [167]. Thus, it remains to be investigated whether NCT impairment leads to α-synuclein accumulation in the nucleus, or if passively diffused nuclear α-synuclein induces impairment of NCT by interacting with Ran.

PD patient-derived induced pluripotent stem cells (iPSCs) with the dominant G2019S mutation in *LRRK2* are reported to show a progressive deterioration in NE morphology, including loss of Lamin B1. The neural stem cells derived from these iPSCs show impaired clonal expansion and compromised differentiation into functional neurons. Moreover, these NE and differentiation defects can be reversed by targeted correction of the G2019S mutation in PD patient iPSCs [171]. NE impairment was also seen in post-mortem human brain samples from both *LRRK2* (G2019S) and idiopathic PD patients [171, 172]. LRRK2 has been shown to translocate to the nucleus, where it directly interacts with Lamin A/C. Both LRRK2 knockdown and G2019S mutant transgenic mice have disorganized nuclear lamina, abnormal NE and leakage of the nuclear protein 53BP1 to the cytoplasm [172]. Another common PD-associated *LRRK2* mutation, R1441C, age-dependently increased NE invaginations and reduced nuclear circularity in conditional transgenic mice. Interestingly, these mice do not show DAergic neurodegeneration, striatal DA deficits, α-synuclein pathology, or motor deficits, implying that aberrant nuclear architecture might constitute an early characteristic of LRRK2-induced neuronal loss in PD [173] (Table 1).

**Table 1:** Summary of nuclear envelop (NE) and nucleocytoplasmic transport (NCT) impairment observed in disease models of Parkinson’s disease (PD)-related genes

PD-linked gene locus	Mutation	NE or NCT alteration	Model system	References
*SNCA*	A53T	Decreased nuclear circularity Increased sensitivity of the nuclear envelope to stress conditions Increased binding and sequestering of Ran	iPSC-derived neurons from PD patients	[168]
	Triplication	Impaired nuclear envelope architecture Decreased nuclear circularity Increased sensitivity of the nuclear envelope to stress conditions Exacerbated nuclear aging	iPSC-derived neurons from PD patients	[168, 171]
*LRRK2*	G2019S	Deteriorated nuclear envelope morphology Reduced Lamin B1 Impaired nuclear envelope Disorganized nuclear lamina Deteriorated nuclear envelope Leakage of 53BP1 to the cytoplasm	PD patient-derived iPSCs Post-mortem human brain Transgenic mouse model	[172] [172, 173] [173]
	R1441C	Increased nuclear envelope invaginations Decreased nuclear circularity	Conditional transgenic mouse model	[174]
	Knockdown	Disorganized nuclear lamina Deteriorated nuclear envelope Leakage of 53BP1 to the cytoplasm	Transgenic mouse model	[173]

The PD-linked E3 ubiquitin ligase, Parkin, has been shown to directly interact with a cytoplasmic filament component of the NPC, Nup358/Ran-binding protein 2. Ubiquitination of Nup358 by Parkin leads to its degradation by the ubiquitin-proteasome system in human neuroblastoma cells, implying that Parkin mutations may compromise Nup358 degradation, causing its accumulation and impairing NCT [174]. To study the role of Nup358 in the stress response, Cho et al. [175] exposed Nup358 haplo-insufficient mice acutely to the Parkinsonian neurotoxin and mitochondrial complex I inhibitor MPTP and observed an exacerbated disease phenotype. Upon MPTP stress, Nup358 haplo-insufficient mice exhibited reduced motor activity, metabolic imbalances, and slower recovery compared to wild-type mice, but notably, they did not have DAergic neuron loss. This study examines gene-environment interactions underlying early Parkinsonism, and more importantly, uncovers the possibility that Nup dysfunction may enhance susceptibility to neurotoxic stress.

## Nuclear pore and nucleocytoplasmic transport impairment under oxidative stress

Increasing evidence from studies on both human brain tissue and animal models suggests that oxidative stress-induced disruptions in the structure and function of NPC are common characteristics of degenerating neurons. Oxidatively damaged proteins accumulate during aging and contribute to cellular dysfunction, particularly in postmitotic cells [176, 177]. D’Angelo et al. [79] have discovered that Nup93 and Nup153 are carbonylated by oxidation of amino acid side chains in aged rat nuclei, which is indicative of oxidative damage. To test the effect of oxidative stress on nuclear leakiness, they treated post-mitotic worms with paraquat and found that paraquat-induced oxidative stress resulted in an increased number and earlier onset of leaky nuclei, which was exacerbated in older worms. In non-neuronal models of oxidative stress, several mechanisms associated with nuclear transport are disrupted, including compromised nuclear membrane integrity, mislocalization of Nups, and altered Ran-GDP/GTP gradient. Oxidative stress has been shown to modulate the expression, stability, and structure of nuclear lamins [178], impacting nuclear membrane integrity and potentially impairing NCT. Being a redox-active protein, Ran may be a prime target of cellular oxidative stress [179]. Ran nucleotide exchange may also be reduced under oxidative stress conditions, thereby disrupting its nucleocytoplasmic gradient [180]. In HeLa cells, hydrogen peroxide-induced oxidative stress leads to the depletion of nuclear Ran and inhibits the docking of import cargo at the NE, blocking classical nuclear import. Nup153 and the nuclear transport receptor importin β also redistribute throughout the nucleus in these cells [181, 182]. Similarly, diethyl maleate-induced oxidative stress reduces the efficiency of classical nuclear import and leads to the mislocalization of multiple Nups in HeLa cells. Specifically, Nup153 and Nup88 are redistributed in the nucleoplasm, Nup88 is mislocalized to the cytoplasm, and Nup50 is more nuclear-retained upon oxidative stress. Additionally, nuclear accumulation of the nuclear transport proteins importin-α and CAS were also identified [183]. In another study, Crampton et al. [184] showed that Ran and Crm1 become increasingly linked, which inhibits nuclear export in HeLa cells exposed to diethyl maleate. The authors also noted that Nup358 gets reduced at the NE and nuclear redistribution of Nup98 occurs. In the above-mentioned studies the nuclear membrane barrier remained intact, so it can be inferred that the oxidative stress-induced changes in nuclear transport are driven by alterations in the transport machinery and not simply a result of NE permeabilization [185]. Mahboubi et al. [186] demonstrated that diethyl maleate also induces the formation of cytoplasmic stress granules that sequester importin-α1, α4, α5, and importin-β1 nuclear transport receptors. Members of the importin α family are involved in transcriptional regulation, and it was found that under normal conditions, importin α1 indirectly associates with poly(A)-RNA, but this interaction decreases in diethyl maleate-induced oxidative stress.

Cellular oxidative stress also induces notable alterations in the phosphorylation and O-linked-N-acetylglucosaminylation (O-GlcNAcylation) patterns of Nups and NCT machinery. Post-translational modifications play a central role in regulating NPC structure and function [187, 188]. Aberrant activation of mitotic kinases that regulate NPC disassembly may lead to defective alteration of NPC components. Indeed, kinases such as ERK, c-Jun N-terminal kinases (JNK), and p38 that are activated and redistributed by ROS have been detected at the NPC [189, 190]. Research from the lab of Gerald Hart [191] shows that oxidative stress induced by hydrogen peroxide and sodium arsenite leads to increased levels of O-GlcNAc transferase and global O-GlcNAcylation in non-neuronal cell cultures. However, in neuroblastoma cells under prolonged oxidative stress, an initial increase and peak in cellular O-GlcNAc levels is followed by normalization of these levels, suggesting that O-GlcNAcylation is highly dynamic in stress responses [192]. In non-neuronal models such as HeLa cells, oxidant-induced signaling activated by diethyl maleate is associated with increased phosphorylation of importin-α1, CAS, Nup153, and Nup88, as well as O-GlcNAcylation of Nup153 in HeLa cells [193]. Crampton et al. [184] demonstrated that in addition to redistribution of Nup98, Nup62, and Nup214, diethyl maleate-induced oxidative stress altered their phosphorylation and O-GlcNAc status. Phosphorylation of these three Nups is increased and O-GlcNAcylation of Nup62 and Nup214 is increased under oxidative stress. These changes in Nup modifications are associated with increased binding of Nup62 to Crm1, Nup153 to Crm1, and reduced interactions between Nup62 and other FG-Nups. In DAergic neurons, O-GlcNAc has been shown to be essential for neuronal survival and synaptic release mechanisms. Selective knockout of O-GlcNAc transferase in DAergic neurons led to an early decrease in axonal arborization and neurodegeneration [194]. However, in PD models, the current research on O-GlcNAcylation has been primarily focused on the inhibitory effect of this modification on α-synuclein aggregation [195, 196]. Since nuclear pore proteins are some of the most abundantly O-GlcNAcylated proteins [197], investigation of the potential alterations in the O-GlcNAc status of Nups in PD is required. Altogether, the studies discussed in this section show that oxidative stress influences NCT and reduces the structural integrity of NPC components. Although most of these studies used non-neuronal oxidative stress models, it can be expected that the molecular pathways leading to NPC and NCT dysfunction may be similar in degenerating neurons under oxidative stress.

## Nuclear pore disruption in other neurodegenerative diseases

### Amyotrophic lateral sclerosis and frontotemporal dementia

Among neurodegenerative diseases, NPC alteration and NCT defects are most extensively characterized in amyotrophic lateral sclerosis (ALS) [18, 198]. ALS symptoms were originally thought to be exclusively motor in nature, but it is now known that cognitive and behavioral problems affect up to 50% of patients, with a smaller subset experiencing significant cognitive impairment and frontotemporal dementia (FTD). Because of their overlap in molecular pathology, FTD and ALS are now considered two ends of a spectrum [199]. Approximately 10% of cases are familial and can be caused by mutations in over 20 currently identified genes, though most cases are sporadic. While hexanucleotide expansions to *C9orf72* are the most common cause of both familial FTD and familial ALS, these mutations are also prevalent in patients with sporadic variants of the disease [198]. Dipeptide repeat proteins (DPRs) resulting from these mutations are toxic in nature and have been used widely in ALS-FTD models, though the mechanisms contributing to their characteristic aggregation remain elusive.

A study using ALS mouse models bearing mutations in the antioxidant enzyme superoxide dismutase 1 (SOD1) by Zhang et al. [200] was the first to demonstrate the abnormal distribution of proteins associated with NCT in the surviving anterior horn cells. In 2015, three independent studies conducted by separate groups further implicated NCT deficits in ALS pathogenesis, though with conflicting findings [8]. While one group validated the toxicity of DPRs at *C9orf72* and identified several NCT-related genes that modified their toxicity [201], they found no abnormalities in RanGAP1 localization, contrary to two other studies published in the same period [202, 203]. These findings led to a subsequent influx of immunohistological studies into NCT alterations in ALS, which were quick to identify mislocalization and cytoplasmic accumulations of various NCT proteins with an emphasis on RanGAP1 alterations. Studies investigating the NPC were limited as they only observed small subsets of individual Nups, often produced findings that were not reproduced, and uncovered little about the structure of the NPC as a whole [198]. However, a recent structured illumination microscopy study assessed 23 different NPC components in ALS patient iPSC-derived neurons and post-mortem tissues expressing *C9orf72* mutations, notably finding eight Nups that were lost in both models. This remarkable observation was accentuated by later findings of the same group, who observed decreases in five of those eight Nups in iPSC-derived neurons derived from patients with sporadic ALS [204]. In both studies, repeat RNA initially decreased the nuclear basket Nup Pom121, which led to reductions in the other implicated Nups and highlighted a separate pathological mechanism distinct from DPR-induced NCT dysfunction. This NPC injury was likely overlooked in other investigations because of their reliance on *Drosophila* and DPR models, which do not express or affect POM121, respectively [198].

TDP43, the protein comprising the hallmark aggregates in ALS, has also been the focus of numerous studies into NCT. Though few patients carry mutations in the *TARDBP* gene encoding TDP43, more than 95% of cases show cytoplasmic mislocalization and hyperphosphorylation of this RNA-binding protein [198, 199]. These cytoplasmic accumulations have been seen to obstruct the morphology of the NE and lead to cytoplasmic mislocalizations of various Nups [8, 198]. It is likely that impaired RNA metabolism, stemming from both TDP43 dysfunction and mutations in another RNA-binding protein, FUS, observed in patients, drives much of this downstream NPC injury [8]. Interestingly, different TDP43 abnormalities generate distinct NPC changes, reflecting the heterogeneity of the complex syndrome [198]. In a mutant-TDP43 *Drosophila* model, loss-of-function mutations in Nup50, Nup93, Nup98-96, Nup107, and Nup214 were all protective against retinal degeneration and locomotor deficits [205]. Nonetheless, the mechanisms behind suppression of neurotoxicity by these Nups are unknown, and it is worth noting that mutant *Drosophila* models do not completely reflect the intricacies of ALS in humans [8, 198].

Aside from *C9orf72*, FUS, and TDP43, alterations to various other genes and proteins implicated in ALS have been investigated in the context of NPC-mediated neurodegeneration [8, 198]. ALS-linked mutations in the small actin-binding protein profilin1 lead to significant disruption of NPC structure and NCT by destabilizing actin [206]. In a SOD1 mutant mouse model, RanGAP1 along with four Nups (Nup205, Nup107, Nup50, and Nup210) showed nuclear precipitation and cytoplasmic mislocalization patterns [207]. Though these results suggest some degree of NPC pathology in SOD1 ALS, the study lacked the functional assays and structural observations needed for a clearer picture of pathology in this variant of the disease. Nonetheless, mutations in *SOD1*, have also been implicated in various other diseases including PD [208].

### Alzheimer’s disease

Alzheimer’s disease (AD) is one of the most common neurodegenerative disorders, accounting for 60–80% of all cases involving dementia-related memory and cognitive decline in patients [209]. This insidious disease is marked by neurofibrillary tangles containing hyperphosphorylated tau and extracellular accumulations of amyloid-beta, though TDP43 and α-synuclein pathology have been observed in patients as well [198, 209, 210]. The presence of neurofibrillary tau tangles in close proximity to the NE suggests the potential for their interference with NPC function and nuclear lamina organization [198]. Indeed, a study by Eftekharzadeh et al. [211] uncovered disturbances in Ran gradient with compromised nuclear import and export in AD, which were mediated by interactions between phospho-tau and the NPC. Specifically, soluble phospho-tau associates with the negatively charged C-terminus of Nup98, which displaces it from the central channel of the NPC into cytoplasmic and perinuclear regions. Once bound to cytoplasmic tau, Nup98 facilitates its fibrillation, leading to RanGAP1 mislocalization and downstream deficits in NCT. Interestingly, these interactions with Nup98 appear to depend solely on tau phosphorylation rather than its fibrillation, which was highlighted by the recovery of Nup98 in the NPC following reductions in soluble phospho-tau via doxycycline treatment. Nup62 was also seen to aggregate to perinuclear regions, though its functional consequences in this context are yet to be discovered. The 414 antibody that detects several FG-Nups besides Nup98 revealed abnormal distributions of these proteins within nuclear invaginations, alongside reduced expression of NPCs along the nuclear membrane, though they did not seem to interact with tau in its fibrillated state. Although this marker detected general abnormalities, two specific FG-Nups (Nup54, Pom121) and one non-FG-Nup (Nup133) did not seem to mislocalize or show reduced expression by themselves, indicating that the NPC undergoes a selective deterioration in AD. A study on NCT proteins in autosomal dominant tau mutant iPSC-derived cortical neurons revealed microtubule-mediated NE abnormalities and NCT disruption. These data suggest that impaired NCT is a pathological mechanism in AD and may also be involved in various neurodegenerative diseases with tau aggregation, such as PD [212].

In AD models, aberrant expression of the nuclear basket Nups Nup153 and Tpr also correlates with a decline in neurogenesis. Nup153 expression is found to be significantly reduced in AD neural stem cells, which decreases its interaction with Sox2, a transcription factor that is crucial for neural stem cell renewal. Nup153 levels also decrease in wild-type neural stem cells exposed to amyloid-β or a nitric oxide donor. Restoration of Nup153 levels in AD neural stem cells by treatment with antioxidant compounds enhanced their proliferation and reinstated their ability to differentiate, leading to increased expression of pro-neuronal genes, a higher proportion of cells expressing neuronal markers, and the adoption of a more mature neuronal phenotype [213]. This supports the notion that oxidative and nitrative stressors can impair NPCs in AD. Abnormal expression of Tpr was observed in Aβ pathology in mouse models of AD and in AD patients. The expression of Tpr influenced the NPC count in primary neural stem/progenitor cells, and dysregulated Tpr expression in hippocampal neural stem cells correlated with changes in the NPC count [214]. These findings reveal that Nup pathology also contributes to neurodegeneration through roles independent of NCT, suggesting that these Nups could serve as promising targets for restoring normal neural stem cell behavior and differentiation in neurodegenerative disorders.

### Huntington’s disease

Huntington’s disease (HD) is a relatively rare neurodegenerative condition primarily recognized by its motor symptoms, though prodromal phases consisting of neurocognitive and psychiatric disturbances are common. This disease is driven by striatal atrophy with some loss in cortical regions, stemming from trinucleotide expansion mutations to the *HTT* (huntingtin) gene [198, 209]. Normally, translocation of huntingtin is mediated via interactions of its N-terminus with Tpr [8]. Mutant huntingtin shows increased phosphorylation at serine-16 of its N-terminus, disrupting its transport and leading to nuclear accumulation. Mutations in huntingtin protein also lead to aggregation of this protein with NPC and NCT components. In mouse models, Nup62, Nup88, RanGAP1, and Gle1 were present in these accumulations, which moved toward perinuclear regions as aging progressed [8, 198]. Remarkably, neuronal death in iPSC-derived neurons and *Drosophila* and mouse models was rescued by overexpression of RanGAP1 and Ran, highlighting the potential role of NPC and NCT dysfunction in HD [8]. Human post-mortem tissues showed a similar mislocalization and co-aggregation of huntingtin with Nup62 and RanGAP1, as observed in mice, though these aggregates were more abundant and smaller in size [198].

O-GlcNAc plays a crucial role in regulating proteins in the brain and is indispensable for neuronal survival and synaptic function [215]. Grima et al. [216] demonstrated that NPC O-GlcNAcylation is significantly reduced in the cortex of an HD mouse model. Moreover, elevation of O-GlcNAc levels by O-GlcNAcase inhibition using thiamet-G, rescued NCT defects and significantly reduced cell death in primary rat cortical neurons transfected with mutant disease-causing huntingtin protein.

### Triple A syndrome

Triple A syndrome is a rare autosomal recessive disorder, characterized by adrenal deficiency, achalasia, and alacrima, though neurological symptoms and nerve atrophy are common in patients [217]. The disease is caused by numerous mutations to the *AAAS* gene, encoding the nuclear pore component ALADIN. These mutations mislocalize the protein from the nuclear core of the NPC into the cytoplasm, without impacting NPC structure or nuclear morphology [8, 218]. Instead, the triple A syndrome pathology seems to be driven exclusively by deficits in ALADIN-mediated NCT. Among these deficits is the observed absence of nuclear FTH1, an iron-storage protein that is protective against oxidative stress and is transported into the nucleus by ALADIN [219]. The nuclear import of aprataxin and DNA Ligase I, two other proteins involved in the repair of single-strand DNA breaks induced by oxidative stress, is also reduced in patient-derived fibroblasts carrying AAAS mutations [220]. Therefore, ALADIN mislocalization leads to an increased vulnerability to oxidative stress which appears sufficient to drive the multi-system disorder.

## Perspectives

Neuronal health and function strongly rely on the maintenance of nuclear integrity and compartmentalization facilitated by NPCs. Strong evidence supports the involvement of NPC and NCT dysfunction in aging and in the pathogenesis of various neurodegenerative diseases. Most studies on NPC and nuclear transport disruption have focused on non-neuronal oxidative stress models or neurodegenerative disease models with pathologic protein aggregates, such as ALS/FTD, AD, and HD models. However, specific alterations of Nups and the mechanisms leading to these alterations are influenced by the neural stressors and neuronal populations involved in disease pathogenesis. Common features shared among these diseases and PD include oxidative stress and the accumulation of insoluble protein aggregates and inclusion bodies in specific brain regions. Although mitochondrial dysfunction has not been directly associated with NPC impairment, independent studies [221, 222] have shown that ALS-linked mutations that disrupt the NPC, such as those in profilin1 and FUS, also lead to mitochondrial dyshomeostasis. Likewise, PD-linked mutations in LRRK2 and α-synuclein that affect NE architecture are also related to mitochondrial dysfunction [223, 224]. We propose a significant but currently underexplored connection exists between mitochondrial impairment and NPC alterations in neurodegeneration. Existing literature suggests that PD-related neurodegeneration is associated with alterations in DAergic neuronal nuclei, characterized by structural damage to the nucleus and mislocalization of crucial transcription factors. Importantly, these nuclear alterations seem to be driven by oxidative stress or α-synuclein pathology. The presence of protein aggregates and pathogenic mutations, coupled with ongoing oxidative stress typical of aging, can accelerate a transition towards a neuropathological state where these protein aggregates, along with ROS, further impair NPC functions. However, this potential pathological mechanism remains significantly understudied in the context of oxidative stress-induced DAergic neurodegeneration in PD. Therefore, we hope that our review highlights the clear imperative for additional exploratory research into the role of NPC and NCT dysfunction as they relate to mitochondrial dysfunction, oxidative stress, and protein aggregation in Parkinsonian and related neurodegeneration.

## Data Availability

Not applicable.

## Abbreviations

NE: Nuclear envelope
NPC: Nuclear pore complex
Nup: Nucleoporin
NCT: Nucleocytoplasmic transport
PD: Parkinson’s disease
DA: Dopamine
FG: phenylalanine-glycine repeats
NLS: Nuclear localization signal
NES: Nuclear export signal
RCC1: Regulator of chromosome condensation 1
ROS: reactive oxygen species
MPTP: 1-methyl-4-phenyl-1,2,3,6-tetrahydropyridine
6-OHDA: 6-hydroxydopamine
PINK1: PTEN-induced kinase 1
CREB: cyclic AMP response element-binding protein
ERK: Extracellular signal-regulated kinase
LRRK2: Leucine-rich repeat kinase 2
O-GlcNAc: O-linked-N-acetylglucosamine
JNK: c-Jun N-terminal Kinase
PGC1α: Peroxisome proliferator-activated receptor γ coactivator 1α
iPSC: Induced pluripotent stem cell
DPR: Dipeptide repeat protein
SOD1: Superoxide dismutase 1
ALS: Amyotrophic lateral sclerosis
FTD: Frontotemporal dementia
FUS: Fused in sarcoma
TDP43: Transactive response DNA binding protein of 43 kDa
AD: Alzheimer’s disease
HD: Huntington’s disease
